# Injury and Healing Response of Healthy Peripheral Arterial Tissue to Intravascular Lithotripsy: A Prospective Animal Study

**DOI:** 10.3389/fcvm.2022.787973

**Published:** 2022-03-28

**Authors:** Feng Liu, Yangyang Ge, Dan Rong, Yating Zhu, Jianhan Yin, Guoyi Sun, Xin Jia, Wei Guo

**Affiliations:** ^1^Department of Vascular and Endovascular Surgery, The First Medical Center, Chinese PLA General Hospital, Beijing, China; ^2^Department of Vascular and Endovascular Surgery, The First Hospital of Hebei Medical University, Shijiazhuang, China; ^3^School of Medicine, Nankai University, Tianjin, China

**Keywords:** intravascular lithotripsy, plain old balloon angioplasty, calcified lesion, shock wave, animal, porcine

## Abstract

**Objectives:**

Intravascular lithotripsy (IVL) is a novel clinical technique for the management of severely calcified lesions. However, the biological effects of shock wave on the healthy arterial tissue have not been demonstrated. The preclinical safety study aimed to investigate the vascular response to IVL shock wave compared to plain old balloon angioplasty (POBA) in porcine peripheral arteries.

**Methods:**

The left and right iliofemoral arterial segments of 16 mini-pigs were subjected to IVL and POBA, respectively. The vascular response was evaluated using quantitative vascular angiography (QVA), light microscopy, and scanning electron microscopy (SEM) at 0, 5, and 28 days.

**Results:**

With the emission of shock wave, adjacent muscle contraction was observed. QVA showed there was no statistically significant difference in percent diameter stenosis and late lumen loss between the two groups. SEM examination showed the endothelial cell layer was intact in both groups at all timepoints. Under light microscopy, no area stenosis was observed. However, IVL shock wave resulted in significantly higher percent area stenosis and intimal area at 28 days. Neointima score showed a trend toward a higher rate in the IVL group, although there was no statistically significant difference at 28 days. There were no statistically significant differences in the scored parameters between groups at all timepoints. However, the parameters of inflammation and neointima showed a trend toward higher scores in the IVL group. After disruption of the internal elastic lamina, the arteries demonstrated significantly neointimal thickening.

**Conclusions:**

The safety and operability of IVL are comparable to POBA. The histological response of healthy arteries to IVL shock wave is mild and sustained. IVL shock wave do not cause serious vascular tissue damage, especially endothelial denudation.

## Introduction

Presence of severe calcified lesions increases procedural complexity in percutaneous peripheral artery intervention. Numerous adjunctive intraluminal devices have been used to modify calcified plaques (1). These approaches modified calcified plaques using direct mechanical force applied to the vascular wall (2). With these procedures, due to lack of selectivity, non-calcified parts of the vascular wall adjacent to calcified plaques were also vulnerable to damage. Damage to vascular structural integrity has been shown to hasten neointimal growth and restenosis (3).

Intravascular lithotripsy (IVL) is a novel technique that relies on the energy carried by shock wave rather than direct mechanical force to disrupt intimal and medial calcium. The efficacy of IVL for heavy peripheral arterial calcification has been described in the literature (4, 5). However, there are minimal data concerning the biological effects following intravascular shock wave application. Understanding the impact of IVL shock wave on the vascular wall is critical for safe clinical implementation of this technology. In this safety study, we aimed to investigate the effect of IVL on vascular tissue using several experimental methodologies.

## Materials and Methods

### IVL Device Description

**Figure 1:**
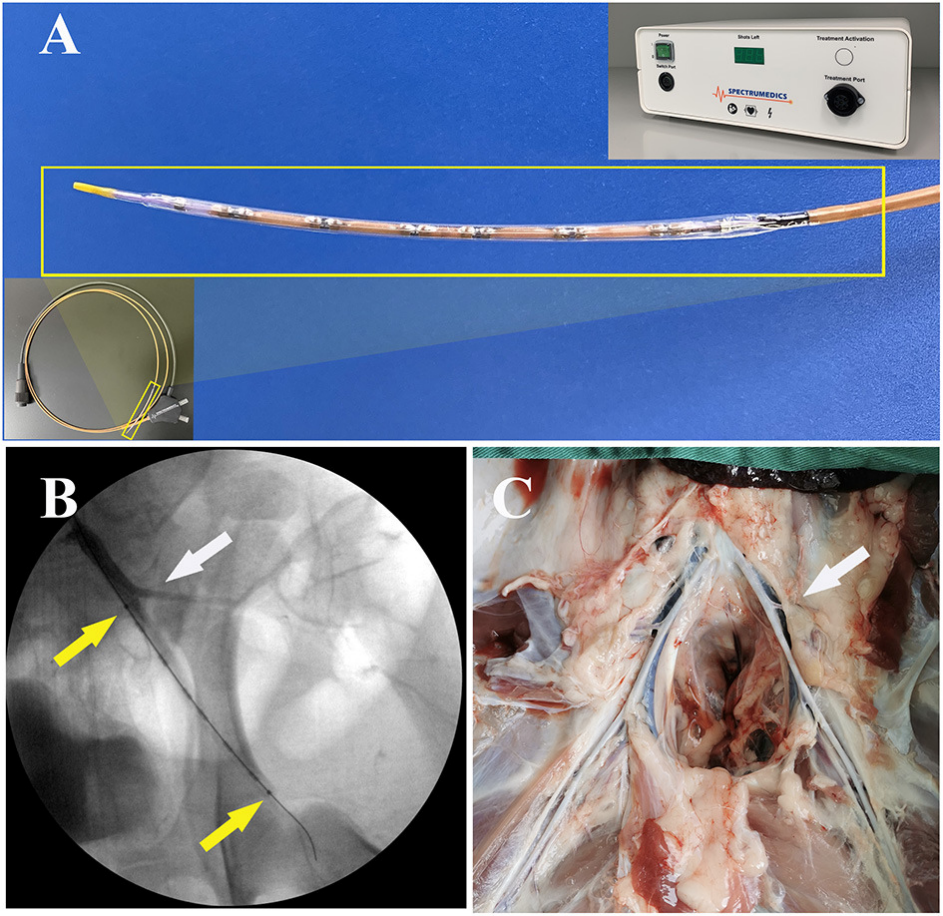
Configuration of the IVL device, angiography during the IVL procedure, and gross observation of the same animal at 28 days postoperatively. **(A)** The IVL catheter with multiple shock wave emitters was mounted in a semi-compliant balloon. Bottom left inset: Overall image of the IVL catheter. Top right inset: Portable control system. **(B)** Markers at both ends of the IVL balloon are clear (yellow arrows). The collateral artery (white arrow) was used to confirm the treated vessel segment location. **(C)** Gross *in situ* observation of treated vessel segments at 28 days postoperatively. The treated arterial segment location was determined by matching with the collateral artery (white arrow). IVL, intravascular lithotripsy.

The IVL device (Sonico-PX, Spectrumedics Medical Technology Co. Ltd., Shanghai, China; energy density, 0.01 mJ/mm^2^; frequency, 1 pulse/second) includes two main components: a semi-compliant balloon catheter (balloon diameter, 4 mm; length, 60 mm; nominal pressure, 6 atm; over-the-wire, 0.014”) with electrohydraulic lithotripsy emitters and a portable control system (Figure 1A). The electrohydraulic lithotripsy emitters in the balloon convert electrical energy into transient circumferential non-focused mechanical shock wave, which passes through the inflated balloon and act on the localized vascular wall. The IVL catheter is connected to the control system by means of a connector cable. Using preprogrammed pulse parameters, the control system automatically delivers the specified shock wave dosage per procedure. The peripheral IVL catheter can deliver 30 pulses per treatment sequence, with a maximum of 180 pulses per catheter. The control catheter in this study used was a semi-compliant plain old balloon angioplasty (POBA) catheter for peripheral arteries (Wanty, Barty Medical, Hangzhou, China; balloon diameter, 4 mm; length, 60 mm; nominal pressure, 6 atm; over-the-wire, 0.035”).

### Experimental Design and Procedures

**Figure 2:**
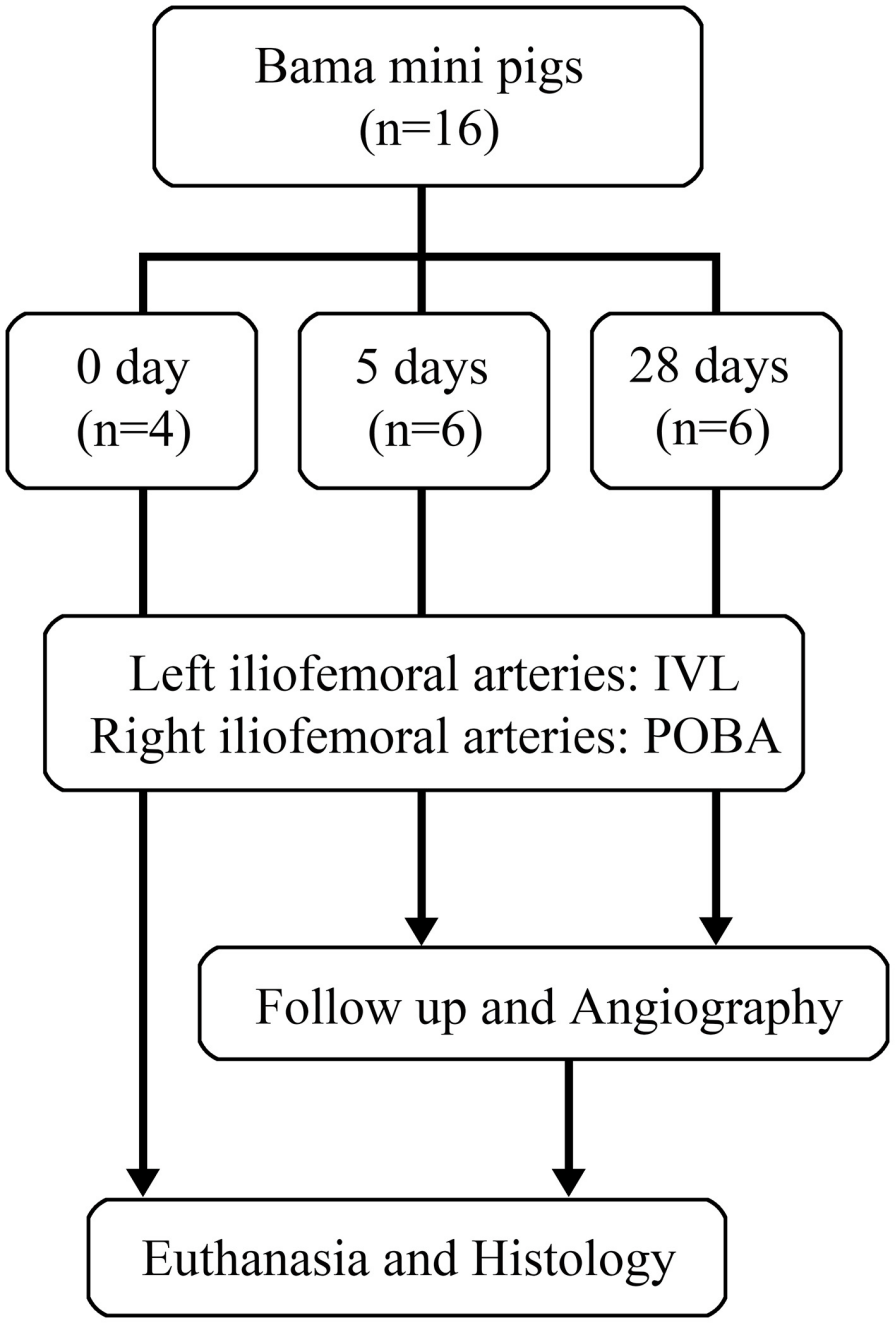
Flowchart of study design. IVL, intravascular lithotripsy; POBA, plain old balloon angioplasty.

The study flowchart is depicted in Figure 2. The study was approved by the Institutional Animal Ethics Committee of Chinese PLA General Hospital. Sixteen Bama mini-pigs (8 months of age; weight range, 24.0–30.5 kg) were included in the study. The left and right iliofemoral arteries of each pig were treated using IVL and POBA, respectively. Four pigs were assigned to the acute group and immediately euthanized after the procedure. The other pigs were sacrificed after undergoing terminal angiography at set time points as follow: 5 days (*n* = 6) and 28 days (*n* = 6). The experiment was performed at an independent animal facility (Thick Biotechnology Co. Ltd., Beijing, China) accredited by the China Food and Drug Administration.

Dual antiplatelet therapy (daily dose of 75 mg clopidogrel and 100 mg aspirin) was initiated at 3 days preoperatively and maintained throughout the study. After sedation had been achieved, all animals underwent intubation and general anesthesia maintenance with inhalation of 2–3% isoflurane. Throughout the procedure, electrocardiography and sphygmomanometry were performed. Endovascular procedures were performed via carotid access. Before catheterization, heparin (400 U/kg) was administered intravenously.

All arterial segments were selected based on quantitative vascular angiography (QVA)-derived reference vessel diameter to achieve a balloon-to-artery ratio of 1.0–1.1:1.0. The IVL balloon was inflated using a diluted (1:1) contrast agent to the nominal pressure (4 atm) to achieve apposition to the arterial wall. After a set of 30 pulses had been delivered, the balloon was deflated and held for 30 s to restore blood flow. These operations were repeated for six cycles to deliver a total of 180 pulses (180 s), which was the maximum number of pulses delivered per IVL catheter. During the procedure, manipulations that could cause vascular injury, such as vessel overstretching and balloon pulling, were avoided. To reduce the influence of osmolarity, isosmotic contrast agents (Visipaque, GE Healthcare Ireland Limited, Dublin, Ireland) were applied for angiography. POBA was performed in the contralateral iliofemoral artery with a balloon-to-artery ratio of 1.0–1.1:1.0. The balloon was inflated for 30 s at the nominal pressure and then deflated for 30 s. The POBA procedure was also repeated for six cycles.

### Angiographic Analysis

All angiographic images were submitted to an independent imaging core laboratory (Jetmed Imaging Core Lab, Beijing, China) for QVA analysis using QAngio-XA Software version 7.3.54.0 (Medis, Medical Imaging Systems, Leiden, Netherlands). QVA analysis was performed by experienced angiographic technicians (certified by the Yale Cardiovascular Research Group) who were blinded to the study design. The treated vessel segments were matched among images collected preoperatively, postoperatively, and during terminal angiography. Matching was performed using collateral branch locations and lengths. The following parameters were measured within the treated vessel segments: minimal luminal diameter (MLD) and mean luminal diameter. Percent diameter stenosis was calculated as follows: (interpolated reference vessel diameter – MLD) ÷ interpolated reference vessel diameter ×100% (6). Late lumen loss (LLL) was calculated as follows: preoperative MLD – postoperative MLD. For better assessment of luminal changes, subsegmental measurement was introduced, dividing the treated vascular segment into three equal subsegments and acquiring individual measurements from each subsegment (Supplementary Figure 1).

### Histological Analysis

Histological changes were observed by light microscopy and scanning electron microscopy (SEM). After gross observation *in situ*, all treated arterial segments were harvested. The locations of treated arterial segments were determined by angiographic landmark matching (Figure 1B). To explore the effects of IVL on adjacent tissues, parallel iliofemoral vein segments and arteries located 2 cm distal to the treated segments were also harvested. The treated arterial segments were cut into three equal portions (proximal, middle, and distal) for histological analysis. Each arterial sample was allocated for the preparation of light microscopy and SEM specimens. The light microscopy specimens were immediately immersed in 10% neutral-buffered formalin and embedded in paraffin using routine procedures. The SEM specimens were immersed in 2.5% glutaraldehyde solution.

#### Light Microscopy Analysis

The light microscopy analysis was conducted in a blinded manner at an independent animal pathology laboratory (PharmaLegacy Pathology Laboratories, Shanghai, China). Specimens were cut into 5-μm sections and stained in a routine manner with hematoxylin and eosin and modified Russell–Movat pentachrome. For histomorphometry, the following cross-sectional parameters were measured using OsteoMeasure (OsteoMetrix, Decatur, GA, USA): external elastic lamina area (EEL), internal elastic lamina (IEL) area, and luminal area. The following parameters were calculated: intimal area = IEL area – luminal area; medial area = EEL area – IEL area; and percent area stenosis = [1 – (luminal area ÷ IEL area)] × 100%.

Histomorphological assessment of the postoperative biological response of vascular tissue was performed using ordinal grading criteria (Supplementary Table 1) that had been modified based on consensus for preclinical stent research (7). The semi-quantitative scoring system incorporated the following parameters: catheter-induced injury, inflammation, neointima, neointimal smooth muscle cell (SMC), endothelial cell coverage, and intimal fibrin. The parameters were scored from 0 to 4 as follows: 0 = none identified, 1 = minimal, 2 = mild, 3 = moderate, and 4 = marked.

#### SEM Analysis

SEM was used to check the integrity and morphology of the endothelial cell layer. After gradient dehydration and critical point drying, the luminal surface was sputter-coated with gold and photographed at low and high magnifications using Quanta 200 SEM (FEI, Hillsboro, OR, USA) at 15 kV. Images of the luminal surface were taken at magnifications of 100× and 500× to check the integrity of the endothelial cell layer. Regions of interest were imaged at incremental magnifications of 1,000× and 4,000× to capture fine details.

### Statistical Analysis

The results are expressed as mean ± standard deviation. To assess the overall statuses of treated segments, the value of each histological parameter was reported as the average of three individual measurements from the proximal, middle, and distal subsegments. Normality was examined using the D'Agostino–Pearson normality test. Normally distributed data were assessed using paired *t*-tests to determine significant differences between the two matched groups; non-normally distributed data were assessed using the Wilcoxon matched-pairs test. Generalized additive mixed models were used to investigate differences in the degree of change between the two groups. Statistical analyses were conducted with GraphPad Prism 6 (GraphPad Software, Inc., La Jolla, CA, USA), as well as the statistical software packages of R (R Foundation for Statistical Computing, Vienna, Austria) and EmpowerStats (X&Y Solution, Inc., Boston, MA, USA). A *P*-value of < 0.05 was considered statistically significant.

## Results

### General Conditions of the Animals

All animals survived to the scheduled study endpoint. Radiopaque markers at the borders of the IVL balloon were clearly visible during the IVL procedure (Figure 1B). Thigh muscle fascicle twitches in ipsilateral limbs could be observed during shock wave emission. There were no significant complications from the IVL and POBA procedures, such as arrhythmia, arterial perforation and dissection, or abrupt closure of arteries and adjacent veins.

### Angiographic Analysis

The QVA findings are summarized in Table 1. The preoperative angiographic variables of the treated vessel segments were comparable between the IVL and POBA groups. Terminal angiography showed that all treated vessel segments remained patent, and no stenosis occurred in the IVL and POBA groups. Angiographic evaluations at 5 and 28 days showed that the MLD and percent diameter stenosis were similar between the IVL and POBA groups. LLL tended to be greater in the POBA group than in the IVL group, but this difference was not statistically significant. The results of subsegmental measurement showed that the MLD and LLL did not significantly differ between the two groups at 5 and 28 days.

**Table 1:** Comparison of quantitative vascular angiography results between intravascular lithotripsy and plain old balloon angioplasty.

	**5 days**				**28 days**			
	**IVL**	**POBA**	***P*** **-value**		**IVL**	**POBA**	***P*** **-value**
	**(*n* = 6)**	**(*n* = 6)**			**(*n* = 6)**	**(*n* = 6)**	
MLD (mm)	3.30 ± 0.60	3.20 ± 0.49	0.625		3.18 ± 0.50	2.92 ± 0.46	0.313
Diameter stenosis (%)	14.40 ± 7.57	13.68 ± 7.81	>0.999		11.47 ± 8.30	11.81 ± 10.20	>0.999
Late lumen loss	0.20 ± 0.49	0.29 ± 0.38	0.844		0.26 ± 0.88	0.49 ± 0.38	0.688
Stenosis rate (%)	0	0			0	0	
**Proximal treated segment**						
Mean LD (mm)	4.31 ± 0.72	4.21 ± 0.90	0.313		4.11 ± 0.48	3.87 ± 0.79	0.844
MLD (mm)	4.13 ± 0.75	4.0 ± 0.94	0.313		3.71 ± 0.56	3.62 ± 0.88	>0.999
Diameter stenosis (%)	5.51 ± 2.95	7.27 ± 2.41	0.563		7.85 ± 5.31	8.89 ± 9.29	>0.999
Late lumen loss	−0.11 ± 0.79	0.47 ± 0.80	0.063		0.411 ± 0.94	0.25 ± 0.59	0.844
**Middle treated segment**						
Mean LD (mm)	4.01 ± 0.66	4.01 ± 0.67	0.781		3.64 ± 0.60	3.37 ± 0.60	0.563
MLD (mm)	3.68 ± 0.68	3.60 ± 0.53	0.750		3.23 ± 0.48	3.09 ± 0.53	0.688
Diameter stenosis (%)	9.25 ± 8.72	6.68 ± 4.97	0.438		15.14 ± 7.15	14.26 ± 8.48	0.563
Late lumen loss	−0.04 ± 0.61	0.18 ± 0.49	0.313		0.51 ± 0.85	0.55 ± 0.40	>0.999
**Distal treated segment**						
Mean LD (mm)	3.75 ± 0.50	3.49 ± 0.50	0.094		3.43 ± 0.48	3.15 ± 0.53	0.478
MLD (mm)	3.39 ± 0.52	3.24 ± 0.53	0.313		3.24 ± 0.51	2.92 ± 0.46	0.219
Diameter stenosis (%)	9.45 ± 6.64	11.56 ± 8.66	0.563		6.42 ± 5.92	9.77 ± 8.43	0.331
Late lumen loss	0.21 ± 0.44	0.27 ± 0.38	0.844		0.21 ± 0.86	0.54 ± 0.41	0.438

*Values are presented as mean ± standard deviation. Diameter stenosis (%) = (interpolated reference vessel diameter – minimal luminal diameter) ÷ interpolated reference vessel diameter × 100%. Stenosis rate is defined as diameter stenosis > 50%. Late lumen loss = preoperative MLD – postoperative MLD*.

*IVL, intravascular lithotripsy; POBA, plain old balloon angioplasty; MLD, minimal luminal diameter; LD, luminal diameter*.

### Histological Analysis

**Figure 3:**
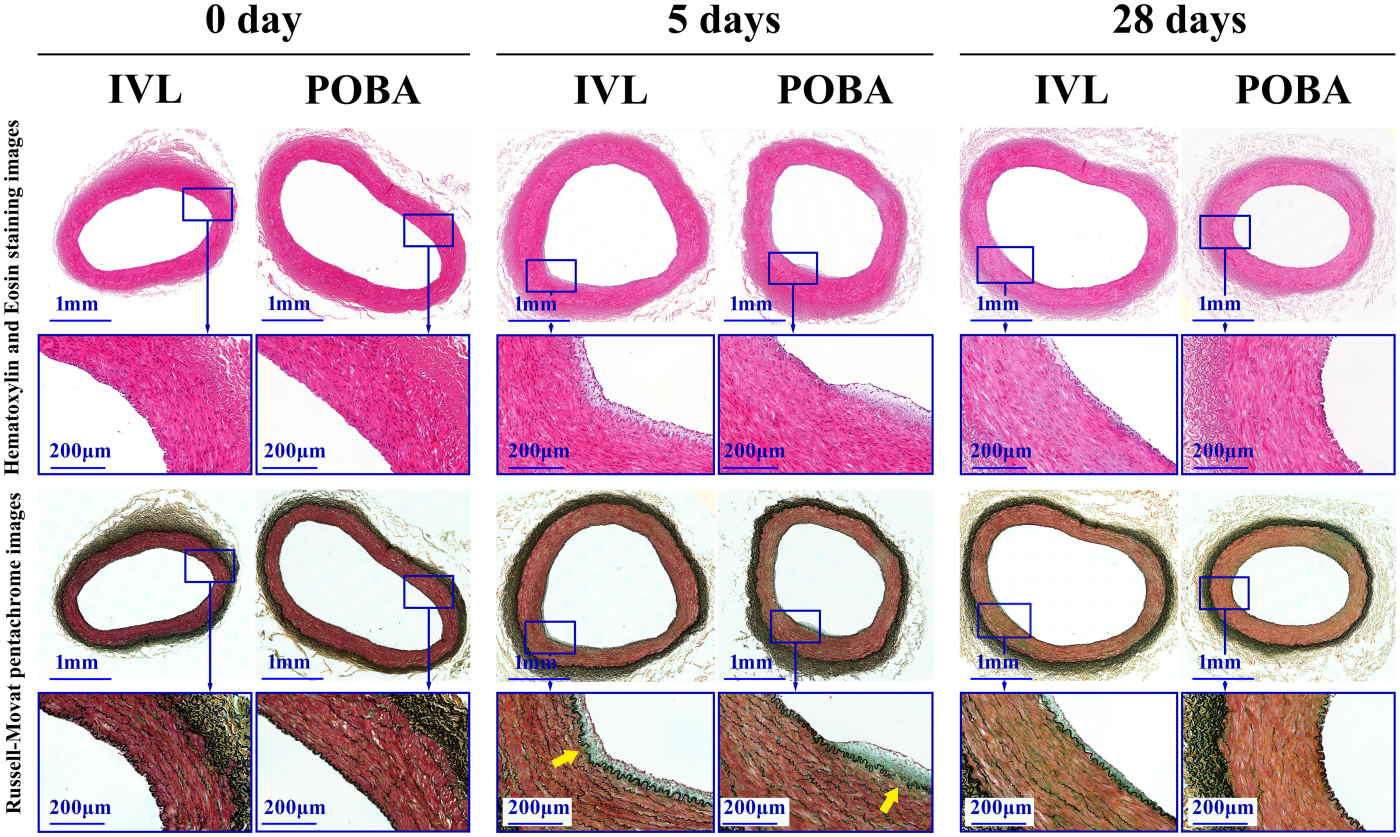
Representative histological images of treated arterial segments at 0, 5, and 28 days postoperatively. Magnified images of rectangular areas are shown below each low-magnification image. Slightly non-uniform neointimal thickening was observed in both groups at 5 and 28 days. Noticeable neointimal thickening was located at sites of internal elastic lamina laceration (yellow arrows). IVL, intravascular lithotripsy; POBA, plain old balloon angioplasty.

Gross observations at all timepoints revealed no hyperemia, petechial hemorrhage, hematoma, or adhesion in the tissues surrounding the shock wave-treated arterial segment (Figure 1C). Light microscopy and SEM showed that arterial wall injury and the inflammatory response caused by shock wave were mild, with no signs of thrombosis or obvious endothelial cell shedding. At 5 and 28 days postoperatively, slightly non-uniform thickening of the neointima was observed in the IVL and POBA groups (Figure 3). Noticeable intimal thickening was evident at the site of internal elastic lamina rupture.

**Figure 4:**
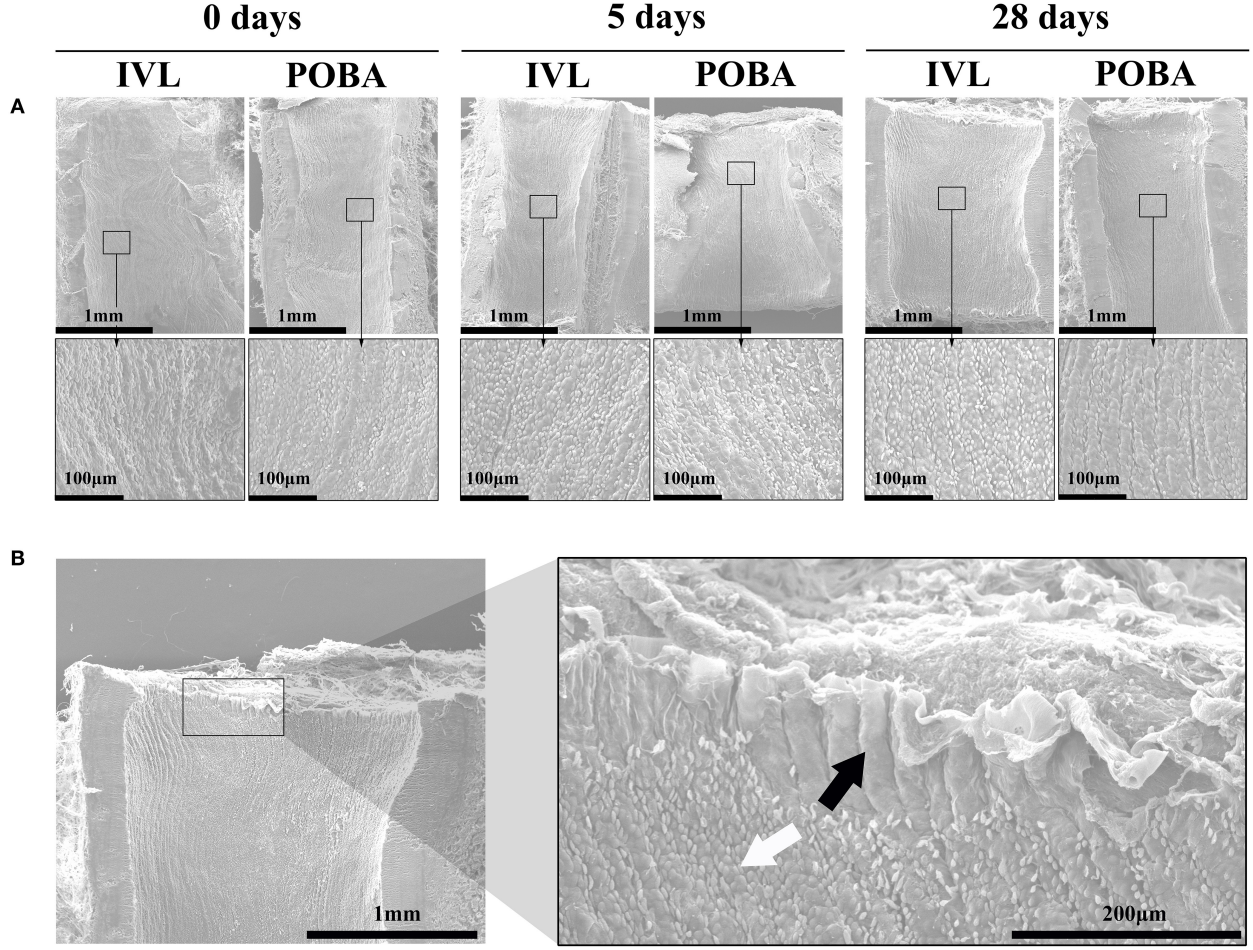
Representative luminal surface SEM images. **(A)** SEM images of the IVL and POBA groups at 0, 5, and 28 days postoperatively. Magnified images of rectangular areas are shown below each low-magnification image. The lumens of both groups were covered by flattened and cobblestone-shaped endothelial cells with a wave-like arrangement. **(B)** The internal elastic lamina (black arrow) was the structural basis of this wave-like arrangement of endothelial cells (white arrow). SEM, scanning electron microscopy; IVL, intravascular lithotripsy; POBA, plain old balloon angioplasty.

There were no obvious histological changes in parallel vein segments or in distal untreated arteries (Supplementary Figure 2). SEM showed that the luminal surface was covered by flattened and cobblestone-shaped endothelial cells with a wave-like arrangement. The IEL was the structural basis of this wave-like arrangement (Figure 4).

The histological results are presented in Table 2. At 28 days, the percent area stenosis and the intimal area were significantly higher in the IVL group than in the POBA group. The medial area was significantly higher in the IVL group than in the POBA group at 5 days.

**Table 2:** Histomorphometric and qualitative histological analyses of entire treated segments.

	**0 day**			**5 days**			**28 days**		
**Entire treated segment**	**IVL**	**POBA**	***P*** **-value**	**IVL**	**POBA**	***P*** **-value**	**IVL**	**POBA**	***P*** **-value**
	**(*n* = 4)**	**(*n* = 4)**		**(*n* = 6)**	**(*n* = 6)**		**(*n* = 6)**	**(*n* = 6)**	
**Histomorphometric parameters**									
Area stenosis (%)	1.07 ± 1.18	0.47 ± 0.43	0.625	3.35 ± 2.26	1.87 ± 0.82	0.156	4.44 ± 2.34	0.90 ± 1.58	0.031
Lumen area (mm^2^)	3.10 ± 0.67	2.89 ± 0.61	0.625	2.65 ± 0.65	2.39 ± 0.50	0.161	2.35 ± 0.51	2.07 ± 0.35	0.688
Intimal area (mm^2^)	0.04 ± 0.05	0.01 ± 0.01	0.625	0.08 ± 0.06	0.04 ± 0.02	0.063	0.10 ± 0.05	0.02 ± 0.04	0.031
Media area (mm^2^)	2.03 ± 0.37	1.91 ± 0.20	0.625	2.15 ± 0.29	1.90 ± 0.415	0.031	2.13 ± 0.25	1.86 ± 0.19	0.219
**Scored parameters**									
Injury (catheter-induced)	1.83 ± 0.58	1.58 ± 0.74	0.750	2.17 ± 0.55	2.28 ± 0.25	0.750	2.11 ± 0.17	2.28 ± 0.44	0.500
Inflammation score	0.08 ± 0.17	0	>0.999	0.50 ± 0.41	0.33 ± 0.30	0.688	0.33 ± 0.37	0	0.125
Neointima	0.33 ± 0.67	0.08 ± 0.17	>0.999	0.94 ± 0.68	0.50 ± 0.35	0.438	1.06 ± 0.33	0.22 ± 0.40	0.060
Neointimal SMC	0	0	NA	0.06 ± 0.14	0	>0.999	0.06 ± 0.14	0	>0.999
Endothelial cell coverage	4.00 ± 0.00	4.00 ± 0.00	NA	3.95 ± 0.14	4.00 ± 0.00	>0.999	4.00 ± 0.00	4.00 ± 0.00	NA
Fibrin in intima	0	0	NA	0.28 ± 0.33	0.11 ± 0.27	0.625	0	0	NA

*Values are mean ± standard deviation of results from three subsegments (proximal, middle, and distal). NA indicates that P-value could not be calculated by paired testing because each row of raw data was identical*.

*IVL, intravascular lithotripsy; POBA, plain old balloon angioplasty; SMC, smooth muscle cell*.

**Figure 5:**
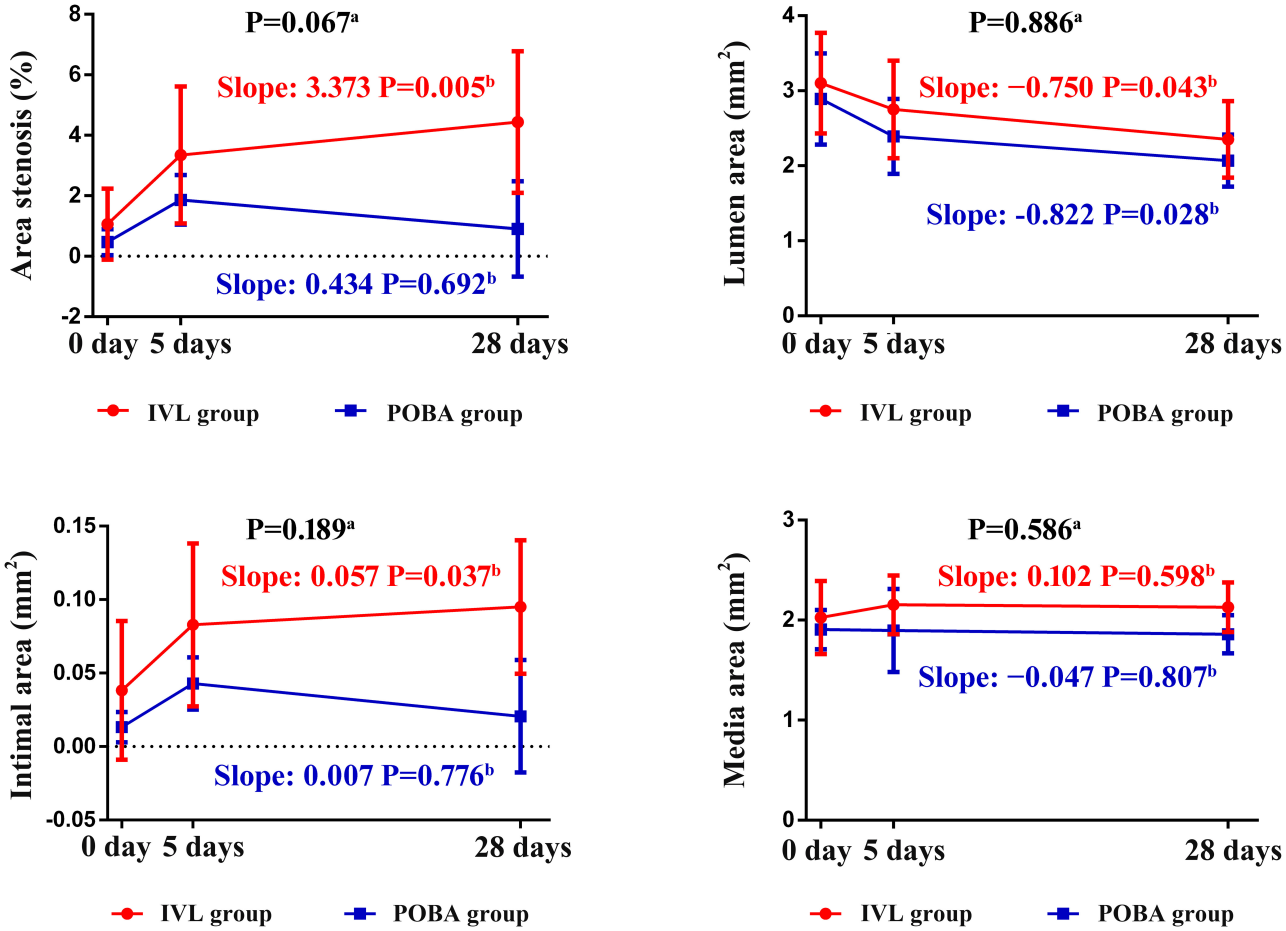
Histomorphometric analyses of treated vessel sections in the IVL and POBA groups. Statistical analysis was performed to compare groups (a) and the change over time (b) from 0 to 28 days using generalized additive mixed models. *P* < 0.05 indicates statistical significance. IVL, intravascular lithotripsy; POBA, plain old balloon angioplasty.

The changes in postoperative vascular remodeling over time are shown in Figure 5. From 0 to 5 days, the percent area stenosis significantly increased [IVL group: slope, 2.284; standard error (SE), 1.085; *P* = 0.045; POBA group: slope, 1.398; SE, 1.085; *P* = 0.209], and the degree of change in both groups was similar (slope difference, 0.886; SE, 1.535; *P* = 0.569). From 0 to 28 days, the percent area stenosis increased significantly in the IVL group (slope, 3.373; SE, 1.085; *P* = 0.005), but showed no significant change in the POBA group (slope, 0.434; SE, 1.085; *P* = 0.692). There was a clear difference in the degree of change between the two groups, although the *P*-value did not reach the threshold for significance (slope difference, 2.939; SE, 1.535; *P* = 0.067).

From 0 to 5 days, the IVL and POBA groups both exhibited a decrease in luminal area (IVL group: slope, −0.351; SE, 0.352; *P* = 0.328; POBA group: slope, −0.499; SE, 0.352; *P* = 0.168) and an increase in intimal area (IVL group: slope, 0.045; SE, 0.026; *P* = 0.096; POBA group: slope, 0.030; SE, 0.026; *P* = 0.260). From 0 to 28 days, both groups showed a significant reduction in luminal area (IVL group: slope, −0.750; SE, 0.352; *P* = 0.043; POBA group: slope, −0.822; SE, 0.352; *P* = 0.028), but the degree of this change was not significantly different between groups (slope difference, 0.072; SE, 0.498; *P* = 0.886). The intimal area significantly increased in the IVL group (slope, 0.057; SE, 2.197; *P* = 0.037), but it showed no significant change in the POBA group (slope, 0.007; SE, 0.026; *P* = 0.776) from 0 to 28 days. The degree of change was similar between the two groups (slope difference, 0.049; SE, 0.037; *P* = 0.189).

With regard to the change in medial area, from 0 to 5 days, the IVL group exhibited an increasing tend (slope, 0.127; SE, 0.192; *P* = 0.328), while the POBA group demonstrated no change (slope, −0.010; SE, 0.192; *P* = 0.958). From 0 to 28 days, the medial area tended to increase in the IVL group, but it remained stable in the POBA group (IVL group: slope, 0.102; SE, 0.192; *P* = 0.598; POBA group: slope, −0.047; SE, 0.192; *P* = 0.807). The change in medial area did not significantly differ between the two groups from 0 to 28 days (slope difference, 0.150; SE, 0.271; *P* = 0.586).

**Figure 6:**
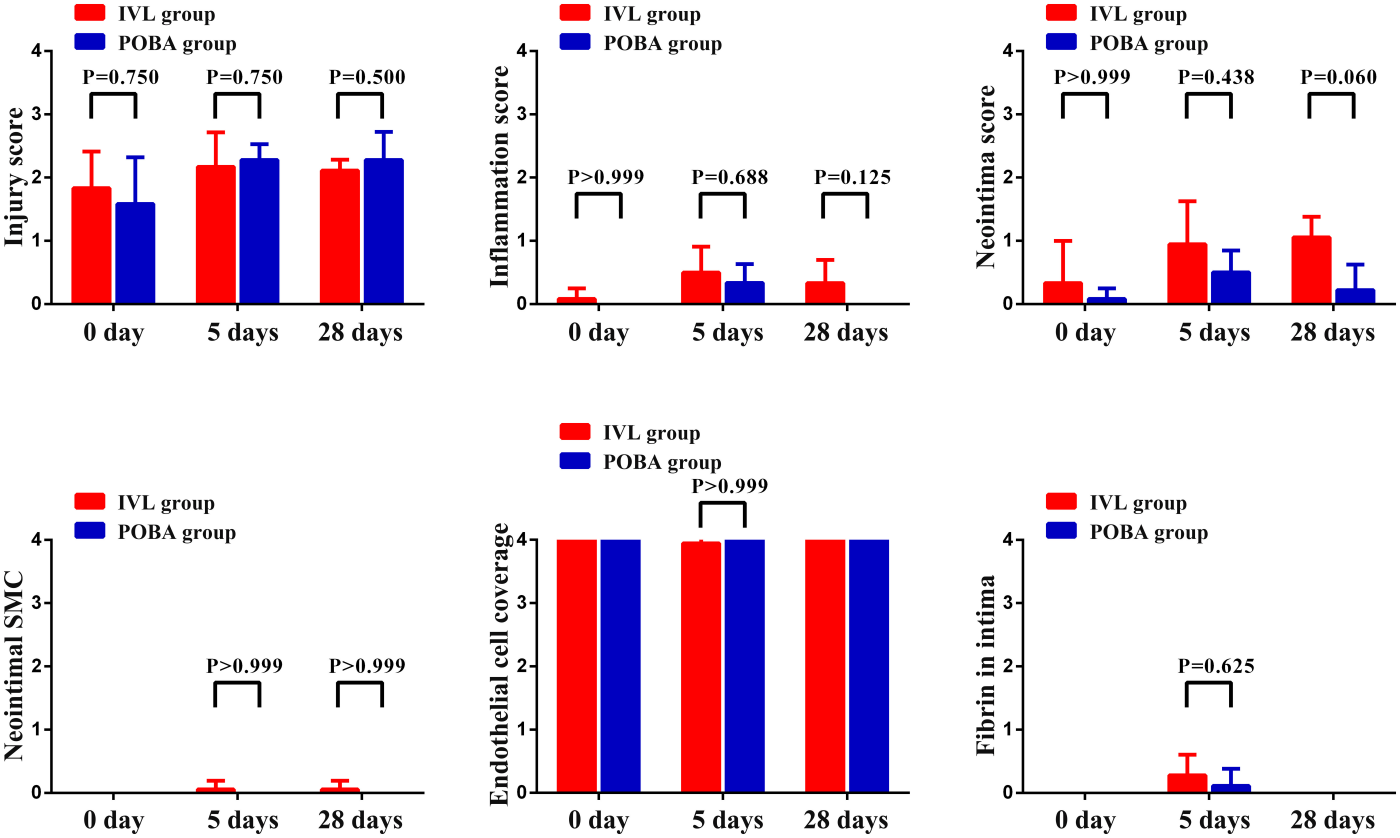
Histomorphological analyses of local vascular tissue responses in IVL and POBA groups. IVL, intravascular lithotripsy; POBA, plain old balloon angioplasty.

The histomorphological scores of the vascular responses to IVL and POBA are presented in Table 2 and Figure 6. Nearly all scores representing injury and healing were very low at all timepoints in both groups, except that the endothelial cell coverage scores were almost always high. There were no statistically significant differences between the two groups at 0, 5, and 28 days postoperatively. However, the inflammatory scores in the IVL group tended to be higher, and this increase was sustained. A greater number of neointimal SMCs were observed in the IVL group than the POBA group at both 5 and 28 days postoperatively. The neointima scores in the IVL group continually increased from 0 to 28 days. This differed from the POBA group, which demonstrated an initial increase and a subsequent decrease (8). In this study, the fibrin score was low in both groups, and fibrin was only observed at 5 days postoperatively, but the score in the IVL group was higher than that in the POBA group.

## Discussion

The main findings are as follows. First, the operability (deliverability, visibility, and balloon dilatation) of the IVL catheter is comparable to that of the POBA catheter. Notably, the process by which the electrohydraulic emitters convert electrical energy into shock wave does not cause electricity-related adverse events (e.g., arrhythmia). Second, the shock wave can propagate through the blood vessel wall and evoke fascicle twitches in adjacent muscles, but there is almost no damage to the vascular endothelium that is directly affected by the shock wave. Third, although the injury and healing responses caused by intravascular shock wave are mild, the shock wave exhibit a tendency to induce a stronger and sustained injury response and healing compared with POBA.

Unlike other devices used for calcium modification, the IVL relies on the pressure wave energy carried by shock waves, rather than the direct mechanical force, to disrupt the calcium. In our study, the muscle fascicle twitches that synchronized with shock wave emission suggested that shock wave energy can propagate through normal vascular wall to stimulate adjacent nerves. Using the whole and subsegmental measurement methods, no significant luminal reduction was found by QVA. The histological results further showed that IVL shock wave hardly damaged the integrity of healthy arterial endothelial cells. The parallel veins and the arteries distal to treated segments were also unaffected. These results support the potential advantages of IVL in calcium modification, that, IVL can possibly avoid early thrombosis and neointimal formation caused by severe damage to non-calcified vascular parts. Additionally, because non-focused shock wave energy transfer decays rapidly with increasing distance through the dispersion medium (9), it can be inferred that the effect of shock wave energy scatter on tissues farther from the IVL balloon should not cause severe damage to surrounding soft tissues.

The histological results showed that shock wave can induce a mild and long-lasting inflammatory response, potentially contributing to neointimal hyperplasia and medial remodeling. There may be several reasons for these changes. First, the intact endothelial cell layer prevents further aggravation of the inflammatory response induced by elastic lamina laceration. Previous studies have confirmed that the continuous inflammatory response caused by vascular injury is the main contributor to neointimal hyperplasia and negative arterial remodeling after angioplasty (3). The intact endothelial cell layer can inhibit proliferation and migration of vascular SMCs, as well as extracellular matrix formation, by preventing growth factor release, platelet activation, and leukocyte adhesion (10–12). Overall, the results of the mild inflammatory response and the small histomorphometric change might be related to endothelial cell integrity postoperatively. Second, shock wave might contribute to intimal–medial thickening and to the inflammatory response at the treated vascular segments through induced biological effects. It has been recognized that in addition to the direct mechanical effect, shock wave energy can produce a variety of biological effects, which can be used in the clinical treatment of chronic ischemia in conditions such as myocardial ischemia (13) and chronic limb ischemia (14). Basic experiments have further confirmed that the shock wave modulates proliferation and metabolism of several cell types in soft tissues via multiple factors and signaling pathways (15, 16). These processes involve induction of Ras-dependent superoxide production (17) and upregulation of extracellular signal-regulated kinase-dependent angiogenic transcription factor (18). These signaling molecules are also involved in vascular SMC proliferation and extracellular matrix formation (19). On the basis of previous observations and reports, we hypothesize that without inducing overt tissue damage, IVL shock wave energy can still induce SMC proliferation and enhance cell metabolism through cytokine signaling, thereby enhancing intimal–medial thickening and vascular wall inflammation. However, the exact molecular mechanism needs further study.

An interesting histological result was observed in this study. Neointimal thickening was evident at sites of IEL laceration. Numerous studies have revealed that injury to the endothelial layer and disruption of deeper vascular wall structures after balloon angioplasty jointly promote SMC proliferation and neointima formation (3). Our histological results suggest that the IEL may be a unique structure such that IEL laceration may lead to activation of other mechanisms of neointima formation. Therefore, maintenance of IEL integrity could potentially reduce neointimal hyperplasia.

This study had some limitations. First, although this animal model has been widely used, its inherent nature means that the results cannot be extrapolated to human clinical treatment. Second, because there are no ideal large-animal models of peripheral arterial calcification, the effects of shock wave energy on calcified vascular tissue require further investigation. There is evident attenuation when shock wave propagates through high-density calcifications, which is similar to ultrasonic wave; thus, it is reasonable to consider that injury to surrounding tissues in the healthy vascular wall would not be worsened when IVL is used to modify calcified lesions. Third, with this relatively small sample size, the possibility that this finding is due to chance cannot be ruled out.

## Conclusions

This animal experiment provided histological evidence for clinical safety of IVL treatment. The results of this preclinical study demonstrate that the safety and operability of IVL are comparable to POBA. The histological response of healthy arteries to shock wave is mild and sustained. IVL shock wave can propagate through the healthy arterial wall without causing serious vascular tissue damage, especially endothelial denudation. -Further investigations are needed to study the impact of IVL shock wave on endothelial and SMC functions in the vascular wall.

## Data Availability Statement

The original contributions presented in the study are included in the article/Supplementary Material, further inquiries can be directed to the corresponding author/s.

## Ethics Statement

The animal study was reviewed and approved by Institutional Animal Ethics Committee of Chinese PLA General Hospital.

## Author Contributions

WG, FL, and YG: conception and design. WG: administrative support. JY and GS: collection and assembly of data. DR and GS: analysis and interpretation of data. DR and FL: drafting of the manuscript. XJ, JY, and YZ: revising it critically for important intellectual content. All authors contributed to the article and approved the submitted version.

## Conflict of Interest

The authors declare that the research was conducted in the absence of any commercial or financial relationships that could be construed as a potential conflict of interest.

## Publisher's Note

All claims expressed in this article are solely those of the authors and do not necessarily represent those of their affiliated organizations, or those of the publisher, the editors and the reviewers. Any product that may be evaluated in this article, or claim that may be made by its manufacturer, is not guaranteed or endorsed by the publisher.

## Supplementary Material

Supplementary Figure 1 Schematic diagram of segmental measurement. P, proximal segment; M, middle segment; D, distal segment.

Supplementary Figure 2 Representative histological images of vein segments parallel to the treated arteries at 0, 5, and 28 days postoperatively. Magnified images of rectangular areas are shown below each low-magnification image. IVL, intravascular lithotripsy; POBA, plain old balloon angioplasty.

Supplementary Table 1 Semi-quantitative scoring criteria of pathologic changes of vessels.
