# Supplementary figures and images for: Injury and Healing Response of Healthy Peripheral Arterial Tissue to Intravascular Lithotripsy: A Prospective Animal Study
Source: Front Cardiovasc Med. 2022 Mar 28;9:787973. doi: 10.3389/fcvm.2022.787973 (PMC8995801; doi:10.3389/fcvm.2022.787973)

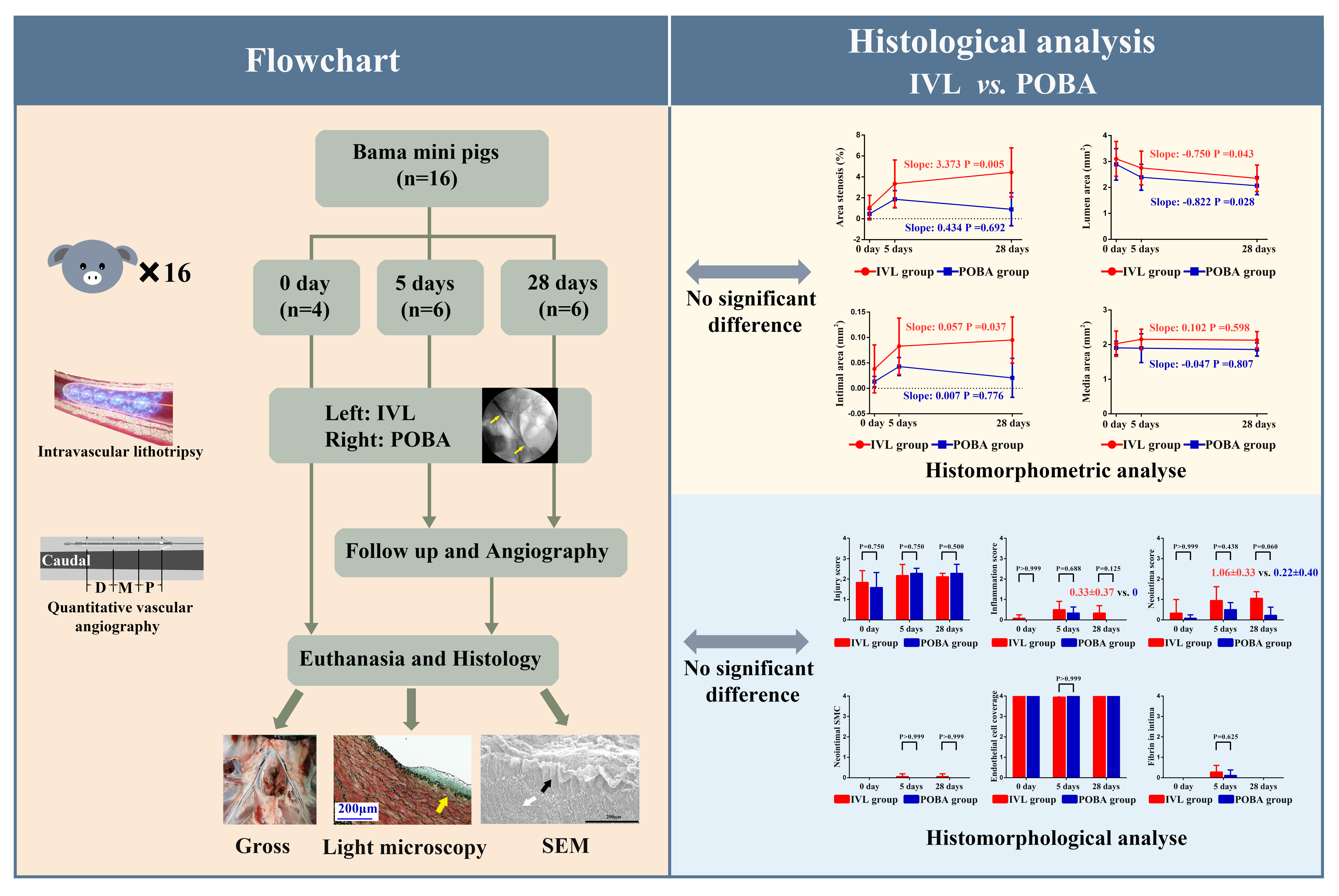

Supplement: Supplementary Figure 1 — Schematic diagram of segmental measurement. P, proximal segment; M, middle segment; D, distal segment. [file Image_1.TIF]

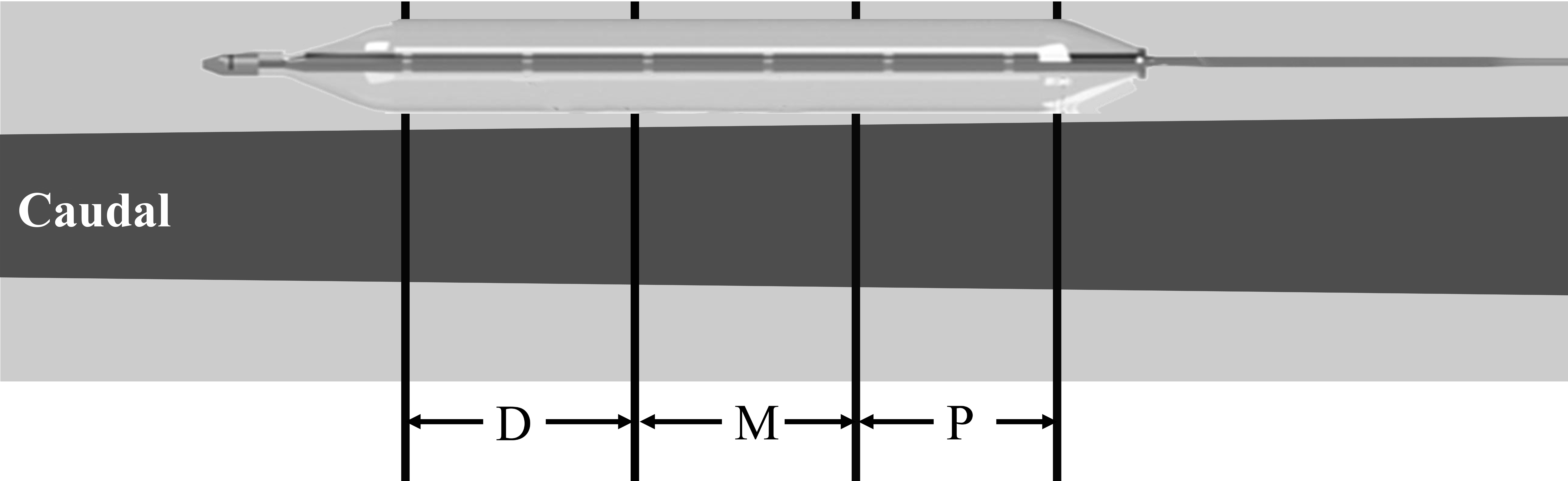

Supplement: Supplementary Figure 2 — Representative histological images of vein segments parallel to the treated arteries at 0, 5, and 28 days postoperatively. Magnified images of rectangular areas are shown below each low-magnification image. IVL, intravascular lithotripsy; POBA, plain old balloon angioplasty. [file Image_2.TIF]
