# Supplementary material for: Injury and Healing Response of Healthy Peripheral Arterial Tissue to Intravascular Lithotripsy: A Prospective Animal Study
Source: Front Cardiovasc Med. 2022 Mar 28;9:787973. doi: 10.3389/fcvm.2022.787973 (PMC8995801; doi:10.3389/fcvm.2022.787973)
Supplement: Supplementary Table 1 — Semi-quantitative scoring criteria of pathologic changes of vessels. [file Table_1.DOCX]

| **Supplemental Table 1.** Semiquantitative scoring criteria of pathologic changes of vessels. | | | | | | |
| --- | --- | --- | --- | --- | --- | --- |
| **Scored Parameter** | **0 (none)** | **1 (minimal)** | **2 (mild)** | **3 (moderate)** | **4 (marked)** |  |
| **Injury (catheter-induced)** | None | Intima lacerated (not extend beyond the IEL) | Media involved, visually lacerated | Separation gap visibly between media and EEL | Adventitia involved, EEL lacerated or separated |  |
|  | | | | | | |
| **Inflammation** | None | Small aggregates of inflammatory cells in adventitia | Mild aggregates of inflammatory cells in adventitia | Multifocal aggregates, or regional infiltration of inflammatory cells in adventitia | Diffuse infiltration of inflammatory cells in adventitia |  |
|  | | | | | | |
| **Neointima** | None | < 25% of the vascular wall thickness | 25%-50% of the vascular wall thickness | 50%-75% of the vascular wall thickness | >75% of the vascular wall thickness |  |
|  | | | | | | |
| **Neointimal SMC** | None | <25 % of neointima | 25-50% of neointima | 51-75% of neointima | >75% of neointima |  |
|  | | | | | | |
| **Endothelial cell coverage** | None | < 25% of the circumference of the arterial lumen | 25-50% of the circumference of the arterial lumen | 50-75% of the circumference of the arterial lumen | >75% of the circumference of the arterial lumen |  |
|  | | | | | | |
| **Fibrin in intima** | None | Small amount, Occasional | Small amount, Multiple foci | Moderate amount, Multiple foci | Marked amount, Multiple or confluent foci |  |
| SMC = smooth muscle cells, IEL = internal elastic lamina, EEL = external elastic lamina | | | | | | |
